# Utilising Interview Methodology to Inform the Development of New Clinical Assessment Tools for Anxiety in Autistic Individuals Who Speak Few or no Words

**DOI:** 10.1007/s10803-022-05509-y

**Published:** 2022-03-18

**Authors:** Georgina Edwards, Joanne Tarver, Lauren Shelley, Megan Bird, Jessica Hughes, Hayley Crawford, Jane Waite

**Affiliations:** 1 Department of Psychology, College of Health and Life Sciences, Aston University, Birmingham, B4 7ET UK; 2 Warwick Medical School, Mental Health and Wellbeing Unit, University of Warwick, Coventry, UK

**Keywords:** Anxiety, Behaviour, Trigger, Assessment, Qualitative

## Abstract

Autistic individuals with intellectual disability who speak few or no words are at high risk of anxiety but are underrepresented in research. This study aimed to describe the presentation of anxiety in this population and discuss implications for the development of assessments. Interviews were conducted with 21 parents/carers of autistic individuals and nine clinicians. Data were analysed using content analysis and interpretative phenomenological analysis. Anxiety behaviours described by parents/carers included increased vocalisation, avoidance and behaviours that challenge. Changes to routine were highlighted as triggering anxiety. Clinicians discussed the importance of identifying an individual’s baseline of behaviour, knowing an individual well and ruling out other forms of distress. This study raises considerations for early identification of anxiety and for subsequent support.

**Supplementary Information:**

The online version contains supplementary material available at 10.1007/s10803-022-05509-y.

## Introduction

Autism is a neurodevelopmental condition associated with reduced verbal language use and high rates of comorbidity with intellectual disability (ID; Maljaars et al., 2012; Matson & Shoemaker, 2009; Mody & Belliveau, 2013). Approximately 50% of autistic individuals have an ID diagnosis (Charman et al., 2011; Loomes et al., 2017) and 25–30% speak few or no words (Anderson et al., 2007; Norrelgen et al., 2015), although rates of up to 50% have been reported (Magiati et al., 2011).

Autistic individuals and individuals with ID are at heightened risk of experiencing anxiety compared to the general population (Costello et al., 2005; Gotham et al., 2013; Green et al., 2015). In autistic individuals, prevalence rates are estimated at 20–42% and research has demonstrated the pervasive and significant impact that anxiety can have on the quality of life of individuals and their families (Adams et al., 2021; Smith et al., 2019). This includes an impact on an individual’s ability to engage in enjoyable activities both inside and outside of the home, classroom performance, social support, and self-esteem (Adams et al., 2021; Smith et al., 2019). Anxiety also has an impact on the wider family, such as parental relationships, a caregiver’s career, ability to attend events with and without the child and caregiver stress (Adams et al., 2021). Autistic individuals and their families, as well as clinicians and professionals have highlighted that better understanding of anxiety is a key priority for autism research, to inform the development of appropriate and effective interventions to reduce anxiety (Cusack & Sterry, 2016).

Despite the increase in research focusing on anxiety in autism in recent years, studies have often focused disproportionately on autistic individuals without ID and/or those who speak in sentences (Jack et al., 2017; Tager-Flusberg & Kasari, 2013). A recent meta-analysis reported that 94% of autistic participants did not have ID and only 2% spoke few or no words, indicating that current research does not accurately reflect the autistic population as a whole (Russell et al., 2019).

One explanation for the current under-representation of autistic people with ID in research may be the challenges of assessing anxiety in those who speak few or no words. These challenges may help to explain the historic lack of validated measures developed for this population. Assessment is complicated by atypical and complex presentations of mental health, difficulties utilising existing self-report measures, diagnostic overshadowing, and behavioural overlap (e.g., disentangling whether a behaviour is indicative of anxiety, autism or pain) (Adams et al., 2019; Hagopian & Jennett, 2008; Lau et al., 2020; Vasa et al., 2016; White et al., 2015). As many as 40% of autistic individuals may meet criteria for impairing anxiety that does not map onto traditional definitions of anxiety using standard diagnostic criteria; anxiety associated with change to routine, novelty, losing access to special interests and unusual specific fears (e.g., balloons, fear of baby crying) have been reported (Hollocks et al., 2019; Kerns et al., 2014; van Steensel et al., 2011).

Several assessment tools have recently been developed to improve the identification of anxiety in autistic populations. Examples include the Anxiety Disorders Interview Schedule with Autism Spectrum Addendum (ADIS/ASA; Kerns et al., 2014), the Anxiety Scale for Autism Spectrum Disorder (ASC-ASD; Rodgers et al., 2016), the Anxiety Scale for Autism-Adults (Rodgers et al., 2020) and the Parent Rated Anxiety Scale for Youth with Autism Spectrum Disorder (PRAS-ASD; Scahill et al., 2019). Additionally, measures have been developed with the inclusion of individuals with ID (Bearss et al., 2016; Toscano et al., 2020). Whilst these measures explore presentations of anxiety that are more specific to autistic populations, individuals with ID only make up a small proportion of the study samples and several items may still require verbal expression of anxiety, making them less appropriate for individuals who speak few or no words. Another recent addition to the literature, the Assessment of Concerning Behavior Scale (ACB; Tarver et al., 2020b) is a screening measure designed to assess broad constructs of mental health in autistic individuals (internalizing and externalizing behaviours). A strength of the ACB is that it is validated in an autistic population with a broad range of abilities. However, the development of specific measures that focus on anxiety in autistic individuals with ID are needed, allowing for a nuanced and thorough understanding of anxiety when internalising difficulties are suspected; and so that the trajectory of anxiety symptomatology in this population can be examined over time.

While the availability of autism-specific assessment tools is improving gradually, there are very few studies focusing on identifying the presentation of anxiety in people with moderate-profound ID who speak few or no words. Flynn et al. (2017) reviewed existing mental health measures for this population, such as the Anxiety, Depression and Mood Scale (ADAMS; Esbensen et al., 2003) and the Diagnostic Assessment for the Severely Handicapped Scale (DASH & DASH-II, Matson et al., 1991, 1995). An identified priority for future research was the confirmation of the validity and reliability of existing measures, as this information was reported for most measures based on a single research study. Additionally, measures were developed from diagnostic criteria for the general population, existing measures that used adapted diagnostic criteria for use with individuals with ID or clinical experience, whereas the authors argue a bottom-up descriptive approach to measure development may be more appropriate in this population. The inadequacy of existing measures may mean that those most at-risk of anxiety are not being identified, preventing early intervention (Costello & Bouras., 2006; Morgan et al., 2008).

Utilising bottom-up interview methodology to describe anxiety presentation can inform the development of the item pool for assessment measures, ensuring that the pool is not restricted to items derived from standard diagnostic criteria for the general population. In addition to examining anxiety presentation in autistic people with moderate-profound ID to improve identification of anxiety, there is also a need to explore the features required in assessment tools to improve their overall utility for this population, e.g., items that aim to minimise diagnostic overshadowing and behavioural overlap.

Due to the identified limitations of existing measures, which may make their application inappropriate for this population, there is an identified gap in the literature and in clinical practice for validated anxiety measures specifically for autistic individuals with moderate-profound ID who speak few or no words (Flynn et al., 2017; Russell et al., 2019). Additionally, there is a paucity of research exploring anxiety presentation in this population and clinician experience of assessment, resulting in a lack of evidence to inform the development of new measures (Adams et al., 2019). There is a need for research to focus on this highlighted gap to document and justify measure development.

Here, we report the findings of a two-stage approach and a combination of qualitative methods, employing a bottom-up interview methodology with parents/carers and clinicians to address the following study aims: *(i)* to explore the presentation of anxiety in autistic individuals who speak few or no words as described by parents/carers and clinicians.; *(ii)* explore clinicians’ experiences of identifying and assessing anxiety in individuals with ID who speak few or no words; (*iii)* identify challenges faced by clinicians when assessing anxiety and the considerations needed to inform the development of assessment tools specifically for anxiety in individuals who speak few or no words. This study was conducted to inform the development of a comprehensive list of items for inclusion in a new assessment measure of anxiety; the validation of this measure against existing anxiety measures will be reported in detail elsewhere. However, we share the detail of our learning in the developmental stage of this project to stimulate the development of other new assessment tools for this population.

## Methods

The current study uses data collected from interviews conducted with parents/carers and clinicians from the first phase of a broader questionnaire development study. Parents/carers and clinicians were included to gain in-depth exploration and insight; they can provide unique perspectives and rich, detailed accounts of behaviour across contexts such as home, school, when out in the community and in clinical settings (Bearss et al., 2016; Cridland et al., 2015; Trembath et al., 2012). Clinician input was also crucial to ensure that information gathered was relevant within clinical practice. This study received a favourable ethical decision from the NHS Research Ethics Committee Wales REC 3 (ref: 18/WA/0139).

### Participants

#### Parents/Carers

Parents/carers were recruited via invitation letters distributed to parents of autistic individuals through an existing participant database held at the University of Birmingham, via social media and the Discover Network run by the charity Autistica. Individual clinical diagnosis of autism was confirmed via parent report, with the majority receiving a diagnosis from a paediatrician (*n* = 13, 62%). All parents/carers included in the analyses reported that their child/the person they care for speaks odd words only or never a word, as assessed by the Wessex Questionnaire (Kushlick et al., 1973). Twenty-one interviews with parents/carers of autistic individuals (*M*_*age*_ = 19.2, *SD* = 11.3, range = 7–52) were conducted. This included nine autistic individuals under 18 years of age (42.9%) and 12 who were 18 years or older (57.1%; see Table 1).

**Table 1:** Demographics for parent/carer interviews

Parent/carer and autistic individual demographics	
Autistic individual mean age in years (SD), *range*	19.2 (11.3), *7–52*
Autistic individual gender	
Male, n (%)	18 (85.7)
Female, n (%)	3 (14.3)
Diagnosis	
Autism, n (%)	21 (100)
Parent/carer gender	
Male, n (%)	1 (4.8)
Female, n (%)	20 (95.2)
Parent/carer mean age (SD), *range*	50.5 (7.2), *37–66*
Relationship to autistic individual	
Mother, n (%)	19 (90.5)
Father, n (%)	1 (4.8)
Sibling, n (%)	1 (4.8)
Household income	
Less than £15,000, n (%)	1 (4.8)
£15,001 to £25,000, n (%)	3 (14.3)
£25,001 to £35,000, n (%)	5 (23.8)
£35,001 to £45,000, n (%)	3 (14.3)
£45,001 to £55,000, n (%)	1 (4.8)
£55,001 to £65,000, n (%)	3 (14.3)
£65,000 or more, n (%)	4 (19)
Not provided, n (%)	1 (4.8)
Highest level of parent/carer education	
No formal qualifications, n (%)	1 (4.8)
Fewer than five GCSEs or O-levels, n (%)	0 (0)
Five or more GCSEs or O-levels, n (%)	2 (9.5)
Three or more A-levels, n (%)	2 (9.5)
University degree, n (%)	6 (28.6)
Masters or Doctoral degree, n (%)	6 (28.6)
Wessex	
Never a word, n (%)	6 (28.6)
Odd words only, n (%)	15 (71.4)
Mean self-help score (SD), *range*^*a*^	6.4 (1.1), *4–9*
SCQ mean score (SD), *range*^*b*^	23.9 (5.7), *13–31*
ADAMS^c^ mean scores (SD), *range*	
General anxiety^d^	11.6 (4.1), *5–20*
Social avoidance	10.4 (4.4), *2–19*
Depressed	4.5 (2.6), *0–8*
Manic/hyperactive^e^	10.4 (3), *3–14*
Compulsive behaviour^f^	5.8 (2.6), *0–9*
Anxiety frequency^g^	
Never, n (%)	0 (0)
At least once a month, n (%)	4 (19)
At least once a week, n (%)	7 (33.3)
At least once a day, n (%)	8 (38.1)
At least once an hour, n (%)	1 (4.8)
Anxiety severity	
Mild, n (%)	1 (4.8)
Moderate, n (%)	7 (33.3)
Severe, n (%)	13 (61.9)

^a^Scores of 3/4/5 are categorised as ‘not able’, scores of 6/7 are categorised as ‘partly able’, scores of 8/9 are categorised as ‘able’

^b^SCQ = Social Communication Questionnaire; cut-off scores of 15 and 20 are suggestive of ‘autism spectrum disorder’ and ‘autism’ respectively

^c^ADAMS = Anxiety, Depression and Mood Scale

^d^Maximum subscale score of 21 for General anxiety, social avoidance and depressed subscales

^e^Maximum subscale score of 15

^f^Maximum subscale score of 9

^g^Anxiety frequency and severity based on parent/carer report in interview. One parent/carer felt unable to provide an accurate response to the question regarding anxiety frequency

#### Clinicians

Clinicians were recruited from participating NHS learning disability clinics providing medical, psychology and nursing services in the West Midlands. Nine interviews were completed with clinicians with a range of roles (psychiatry, clinical psychology, nursing, paediatrics) and clinics. The average years of experience working in clinical services supporting individuals with autism and/or ID was 16 years (*SD* = 10.48, range = 5–36 years). Participant demographics are shown in Table 2.

**Table 2:** Demographics for clinician interviews

Clinician participant demographics	
Gender	
Female	6
Male	3
Clinician role	
Clinical Psychologist	2
Community Learning Disability Nurse	2
Clinical Nurse Specialist	2
Consultant Paediatric Neuropsychiatrist	1
Consultant Paediatric Liaison Psychiatrist	1
Consultant Community Paediatrician	1

### Procedure

#### Parents/Carers

Parents/carers responding to study adverts and invitation letters were invited to complete study consent forms and questionnaires online (for sample characterisation; see below). Following this, a member of the research team arranged a date and time with parents/carers to complete the interview. Due to the national recruitment approach for parent/carer recruitment, interviews were completed over the telephone. Interviews were conducted by a member of the research team (GE or JT); interviewers were trained prior to data collection to ensure consistent interview style. With parental consent, parent interviews were audio-recorded and were transcribed verbatim. Two (9.5%) parents/carers did not consent to recording, so an interview coding document was used during these interviews to identify the presentation of anxiety for these individuals. Interview length varied from 25–149 min with a mean of 58 min (*SD* = 30.4).

The main aim of the parent/carer interviews was to identify the presentation of anxiety in autistic individuals including behaviours associated with anxiety and triggers for anxiety to help inform the development of the item pool for a new measure. Parents/carers were also asked to discuss the frequency and severity of anxiety experienced by their child/the person they care for (See Table 1). The interview schedule was previously developed as part of a research project aiming to explore anxiety in ID utilising clinical formulation frameworks (Royston et al., 2021). Example interview questions are provided in Table 3.

**Table 3:** Example interview questions (parents/carers)

Parent/carer interview question examples
Can you describe a recent example or period of time when (X) showed this anxiety?
When (X) feels anxious, how do they behave during this time?
Does the onset of anxiety seem to be linked to any triggers or causes?
*Example question prompts*
Do you see any physical changes in their bodies?
Do you see changes in how the person moves?
Do you see any changes in their face?
Have you noticed any patterns when anxiety occurs?

#### Clinicians

Clinician interviews were designed to supplement parent/carer interviews regarding the presentation of anxiety. In addition, clinician interviews aimed to explore current methods and challenges of assessing anxiety in clinical services to highlight important considerations for the development of assessment tools to assess mental health in individuals who speak few or no words. Clinician interviews were conducted face to face (by GE) at the clinicians’ usual place of work and were recorded and transcribed verbatim. The mean duration of these interviews was 55.89 min (*SD* = 10.67, range = 33–70 min). The interview schedule was developed for the current study, mirroring questions on the parent/carer interview. Additional questions were asked to explore methods of assessing anxiety and associated challenges. Example questions are provided in Table 4.

**Table 4:** Example interview questions (clinicians)

Clinician interview question examples
When you are assessing anxiety, are there specific things you look for in minimally verbal people?
When you are assessing anxiety in minimally verbal people, how do you differentiate this from other diagnoses, such as pain or autism spectrum disorder?
Do you ever think anxiety is diagnosed incorrectly, or overlooked? What leads to this?
*Example question prompts*
Do you see any physical changes in their bodies?
Do you see changes in how the person moves?
Are there particular behaviours that are shown that cause confusion?

### Measures

Parents/carers completed the following questionnaire measures to characterise the sample of autistic individuals.

#### The Wessex Questionnaire

The Wessex Questionnaire (Kushlick et al., 1973) assesses an individual’s social and physical adaptive ability (e.g., speech, self-help ability). The questionnaire has 16 items and can be used as a proxy measure of ability, with higher scores indicating greater ability. Inter-rater reliability is good at 0.62 (Kushlick et al., 1973; Palmer & Jenkins, 1982).

#### Social Communication Questionnaire

The Social Communication Questionnaire (SCQ; Rutter et al., 2003) is a screening tool for ASD characteristics. It has 40 items, and scores are summed to provide a total score with a high score indicating more ASD characteristics. The SCQ is suitable for individuals with ID (Berument et al., 1999) with good internal consistency for verbal and non-verbal individuals (α = 0.94 and α = 0.89 respectively; Marvin et al., 2017).

#### Anxiety, Depression and Mood Scale

The Anxiety, Depression and Mood Scale (ADAMS; Esbensen et al., 2003) explores behaviours related to anxiety, depression, and mania. There are 28 items rated on a Likert scale of 0 ‘not a problem’ to 3 ‘severe problem.’ The questionnaire is validated with informants of individuals with mild to profound ID. Test–retest reliability and internal consistency are excellent at 0.81 and 0.80 respectively (Esbensen et al., 2003).

### Data Analysis

The manuscript presents the combination of content analysis for parent/carer and clinician responses to highlight and quantify the behaviours and triggers associated with anxiety. Further analysis was conducted for clinician responses, in the form of Interpretative phenomenological analysis (IPA) to explore clinician experience of assessing anxiety, associated challenges and considerations needed for the development of new assessment measures. The use of a combination of analysis techniques to make sense of qualitative data has been demonstrated in autism research previously (Brown et al., 2020). Each analysis approach is described below.

#### Content Analysis to Describe Anxiety Presentation

For all analyses, transcriptions were analysed using NVivo 12 (QSR International UK, 2018). To fulfil the first aim of the study, content analysis was used to identify codes in the parent/carer and clinician narratives to quantify behaviours associated with, or attributed to, anxiety. Information related to triggers for anxiety was also coded. A manifest analysis was chosen to ensure that findings remained true to the text and to code exact words used by participants, with limited interpretation from the researcher (Bengtsson, 2016). First, interview transcriptions were read several times to obtain familiarisation with the data. The text was then divided into meaning units, where the text relating to the presentation of anxiety was highlighted. The meaning units were then organised into codes and categories. A code is a descriptive label given to a meaning unit (e.g., hypervigilance). Codes that were related to each other were grouped together to form the categories of anxiety behaviours and triggers (Erlingsson & Brysiewicz, 2017). For example, proximity seeking was coded as an anxiety behaviour and busy environment was coded as an anxiety trigger (See Table 5 and 6 for further examples and endorsement across autistic children vs. adults).

**Table 5:** Content analysis findings: behaviours associated with/attributed to anxiety reported by parents/carers (broken down to indicate endorsement by parents/carers for autistic children vs. adults) and clinicians

Behaviours associated with anxiety	Number of parents with code (%) [n/% child]	Number of clinicians with code (%)	Example codes	Quote example from parent/carer	Quote example from clinician
Low order repetitive behaviour or stimming	10 (47.6) [3/30]	3 (33.3)	Hand flapping, rocking, bouncing	*“there’s a rocking… it’s not sort of calm rocking, it’s distressed rocking”*	*“stereotypical behaviours, rocking”*
Higher order repetitive behaviours or OCD-like behaviours	10 (47.6) [4/40]	4 (44.4)	Closing windows and doors, wanting thing straight	*“much more needing to control the environment around him”*	*“trying to exert control over the environment… insisting on having certain things a certain thing”*
Repetitive speech/asking	12 (57.1) [7/58.3]	1 (11.1)	“home, home, home”, “no, no, no”	*“repetitive asking, requesting”*	*“even those with very low verbal skills might repeat whatever word they do know over and over again”*
Avoidance/refusal patterns	19 (90.5) [8/42.1]	9 (100)	Running away from something, not wanting to get out of car, hide behind arm, need to flee	*“he would perhaps try to run away”* *“his only way to express what he is feeling is to refuse to cooperate”*	*“tried to run away”*
Freezing/rooted to the spot	4 (19) [1/25]	0 (0)	Won’t move from spot, physically getting stuck	*“he will plant himself… he will almost be quite rooted to the spot”*	*N/A*
Withdrawal/reduced vocalisation/shutting down	12 (57.1) [6/50]	7 (77.7)	Reduced ability to communicate, quiet, withdraws	*“he internalises when he’s anxious about something… he doesn’t say it, he will just kind of step back”*	*“they might just absolutely go in themselves and just not want to communicate with the world”*
Increased vocalisation/communication	20 (95.2) [9/45]	8 (88.8)	Shouting, screaming, crying, groaning, moan, grumble, whimper	*“he will shout out or maybe scream… where you hear a groan noise, like a low rumble noise”*	*“an increase in cry-like sounds and a grunting or just any type of negative vocalisations”*
Facial expression/change	17 (81) [8/47.1]	6 (66.6)	Frown, grimace, blank, tense, worried, tighten lips	*“you can see it in her face, she looks anxious and agitated, sort of tense”*	*“you can see from their facial expression that they’re concerned and they’re not at ease”*
Body changes/movement	18 (85.7) [8/44.4]	6 (66.6)	Tense body, pacing, sweating, trembling, shaking, breathing changes, rigid	*“his heart is pounding, he’s profusely sweating”* *“he’ll start pacing up and down the room”*	*“increased sweating, increased heart rate, increased breathing”*
Behaviours that challenge	19 (90.5) [9/47.4]	9 (100)	Hitting, biting, pushing, kicking self or others, throwing or damaging things	*“bites his hand or he hits his head”* *“he will kick things… doors, walls, windows”*	*“an increase in self-injury or an increase in aggression or destruction”*
Proximity seeking	9 (42.9) [3/33.3]	2 (22.2)	Clinging, following, touching, holding onto others	*“he started to become a bit clingy… he would stick to somebody like glue”*	*“apparent increased need or wanting of attention from the main caregiver”*
Hypervigilance	3 (14.3) [0/0]	0 (0)	Looking around, watching, on edge	*“she’s really honing in, you can see her… she’s watching around… she can’t focus on what to eat because she’s too worried about the environment”*	*N/A*
Change in overall demeanour	14 (66.7) [7/50]	6 (66.6)	Agitated, frustrated, not so relaxed	*“I can pick up from her tension. You can just tell. She’s not as relaxed”*	*“he becomes agitated”, “just a general change in mood”*
Change in everyday behaviour/skills	4 (19) [1/25]	2 (22.2)	Regression in skills, defecating, wetting, vomiting	*“when his anxieties are high all that goes out the window, his communication goes back to where it was all those years ago”*	*“regression as well in skills, so maybe being less functionally able or appearing that way”*
Covers ears	3 (14.3) [2/66.7]	0 (0)	Puts hands/sticking fingers in ears	*“he will put his hands over his ears, he wants to sort of separate himself”*	*N/A*
Hyperactivity	7 (33.3) [4/57.1]	4 (44.4)	Unable to sit down/sit still, climbing, on the go	*“be unable to sit down, sit still, he’d be constantly climbing, constantly on the go”*	*“you can often see that they can’t settle… they’re circling the room”*
Changes in food habits	8 (38.1) [2/25]	5 (55.5)	Won’t eat, regurgitation, eating too quickly	*“has an impact on how he eats, because he eats too quickly or too slowly”* *“he won’t eat anything”*	*“you’ve got to consider appetite… either overeating maybe or not eating enough”*
Changes in sleep habits	3 (14.3) [0/0]	4 (44.4)	Couldn’t sleep, scared to sleep	*“he was scared to go to sleep”*	*“somatic changes are indicators of anxiety… sleep disturbance”*
Covering self	2 (9.5) [0/0]	0 (0)	Wrap/cover self in curtain, jumper, blanket	*“he then covers himself up with a jumper, or a blanket, or a cushion”*	*N/A*
Biting scarf	1 (4.8) [0/0]	0 (0)		*“she always wears a scarf so she can bite into the scarf”*	*N/A*
Drawing	1 (4.8) [0/0]	0 (0)		*“he spends a lot of time drawing things and he will repeat he’ll like create an exercise book out of sheets of paper and do about 40 drawings of a van”*	*N/A*
Non-epileptic seizure presentation	1 (4.8) [0/0]	0 (0)	Crawl around, lie down, shaking, trembling, can’t walk, eyes flicker	*“her anxiety manifests itself as what we think are what are called non-epileptic seizures… she’s physically incapacitated whilst that is going on”*	*N/A*

**Table 6:** Content analysis findings: anxiety triggers reported by parents/carers (broken down to indicate endorsement by parents/carers for autistic children vs. adults) and clinicians

Anxiety trigger	Number of parents with code (%) [n/% child]	Number of clinicians with code (%)	Examples	Quote example from parent/carer	Quote example from clinician
Fear of what other’s think/getting into trouble/upsetting others	4 (19) [2/50]	0 (0)	Very sensitive to being liked, thinks he’s being told off	*“he thinks he’s done something wrong, he thinks he’s being told off”*	*N/A*
Communication frustration/difficulty	7 (33.3) [5/71.4]	2 (22.2)	Can’t always communicate needs, she really wants to communicate	*“sometimes she really wants to communicate and she can’t really communicate, I think she can probably understand more than she can voice”*	*“frustration around not being able to communicate his needs effectively”*
Unpredictability/uncertainty	16 (76.2) [5/31.3]	7 (77.8)	Not knowing what’s happening, doesn’t understand, dogs, young children, cats	*“I think it’s the unpredictability of the outside world that feeds his anxiety”*	*“not really knowing what’s happening, what the plan is for them, in terms of the day and things”*
New/unfamiliar situations or people	10 (47.6) [4/40]	3 (33.3)	New teachers, students, unfamiliar children or adults, new staff, lack of trust	*“new experiences cause her huge anxiety”* *“anybody that is new that he doesn’t know”*	*“new member of staff started working with this person…we started seeing an increase in behaviour”*
Needs not being met	11 (52.4) [6/54.5]	4 (44.4)	Not having enough support, not able to do something that he wants to do	*“he didn’t feel supported, they didn’t understand him”*	*“coming towards the end of primary when perhaps the child might have outgrown or the schools no longer meeting their needs”*
Demands/not meeting demands	10 (47.6) [5/50]	5 (55.6)	If he doesn’t want to do something, social demand, questions, choices	*“whether it is demand of an interaction…a task…to do something that wasn’t quite on his agenda at that moment”*	*“the expectations of them are massive because they’re in college and just getting people to strip back and work with them on a more functional level helps”*
Absorbing tension in environment	7 (33.3) [3/42.9]	0 (0)	Other people upset around him, he doesn’t like slight tension in the room, if voices are raised	*“if people sort of shout…she seems to pick up the vibe of the atmosphere”*	*N/A*
Changes to routine/expectation	16 (76.2) [5/31.3]	9 (100)	Lots of staff changes, Christmas because so many changes, changes from the norm, summer holidays	*“any change in routine seems to majorly unsettle him especially in his own environment”*	*“are there any changes going on at the moment, has anything been done differently in terms of change to teacher to cause anxiety, we’re immediately kind of thinking”*
Too much structure/knowing plans too far in advance	1 (4.8) [0/0]	1 (11.1)	Her anxiety rises too far in advance, he’s constantly asking questions	*“if she knows too far in advance, her levels of anxiety appear to rise”*	*“as soon as you start preparing him for something he’s constantly asking questions…starts getting anxious”*
Busy environments	9 (42.9) [3/33.3]	2 (22.2)	Crowds, lots of people, shopping centres, busy lobby, cafés, pubs	*“we can’t take him on a normal school holiday… it’s way too busy, he doesn’t like crowds”*	*“does not like crowds or busy places”*
Loud environments/particular noises	14 (66.7) [5/35.7]	5 (55.6)	Places that are loud, people that are loud, too noisy, any kind of noise, very sensitive to noise	*“if people are loud or shouty around her”*	*“the noise, that was a real issue for him where hitting just went right up”*
Particular places, situations, stimuli or people	12 (57.1) [4/33.3]	7 (77.8)	Dentist, appointments, hospitals, balloons, pigeons	*“he’s never liked going to appointments…I’ve always had his dentist going to him”*	*“birds is another one…balloons because they didn’t like the movement of the balloon”*
Overwhelmed by sensory experiences/too many activities	14 (66.7) [5/35.7]	6 (66.7)	Sensory overload, sounds, lights, tactile defensive, lots of sensory stimuli	*“if that sensory mode gets too overloaded, that’s where the coping mechanism goes”*	*“sensory overload is a massive thing”*
Under stimulated/bored/sensory seeking	4 (19) [2/50]	3 (33.3)	Under stimulation, linked to sensory needs, opportunity to express and release that	*“trying to not let him be bored or left unattended”*	*“under-stimulation can have a bearing upon, their levels of anxiety”*
Sensory difficulty (direction not stated)	0 (0) [0/0]	1 (11.1)		*N/A*	*“environmental type stuff, it could be linked to like sensory needs”*
Social interactions/meeting people	4 (19) [2/50]	0 (0)	She doesn’t interact well with people, risk of social interaction	*“the risk of interaction, the fear of what might be expected”*	*N/A*
Transitions	6 (28.6) [3/50]	4 (44.4)	Transitioning between rooms, from home to school and home again, primary to secondary school	*“transitioning into school or from school back home or just moving into the dining area”*	*“we get spikes of referrals at key transition times…in October when schools have been back a month”*
Enclosed spaces	2 (9.5) [1/50]	0 (0)	Doesn’t like small places, claustrophobic	*“he doesn’t like being in enclosed spaces”*	*N/A*
Setting events	14 (66.7) [6/42.9]	7 (77.8)	Tired, pain, cold, hot, hunger, hormonal, menstruation, physical health difficulty, epilepsy, medication change/effects	*“things like hunger or pain or tiredness may well influence that”*	*“could be pain or some physical health syndrome but actually it is giving them anxiety because they can’t understand what’s happening in their body”*
Unable to fix issue	2 (9.5) [2/100]	0 (0)	Unable to fix problem, DVD stuck, iPad frozen	*“it’s just ‘fix this’…through desperation”*	*N/A*
Insistence on sameness	5 (23.8) [2/40]	1 (11.1)	Seat belt wasn’t on right, deviation from rigidity, people in certain positions	*“he likes it a certain way…he’s just decided that’s his thing…it certainly causes anxiety”*	*“being very rigid about how you want things to be done and when that’s deviated from they display anxiety”*
Removal of interests	3 (14.3) [2/66.7]	2 (22.2)	Worry that someone will take it, run out of bubbles, not being able to access special interest	*“I am anxious because I know my bubbles have nearly run out and I need to get some more”*	*“not being able to access their special interest or they have a particular restricted interest which means they aren’t able to access that particular interest as much”*
Worry after hurting others	1 (4.8) [1/100]	0 (0)	Trying to rub away blood from scratch	*“if it bleeds, you can see the anxiety, he tries to rub it out, ‘go away, sore gone”*	*N/A*
Conscious of own limitations	1 (4.8) [1/100]	0 (0)	Feels different to others, aware of difficulties	*“he is more aware of his difficulties as in like his language and interaction skills”*	*N/A*
Missing out/not being part of activity	1 (4.8) [1/100]	0 (0)		*“he was feeling isolated that we were able to communicate in a way that he can’t”*	*N/A*
The way others treat person	4 (19) [0/0]	0 (0)	They weren’t very nice to him, some of the staff were getting bossy like ‘do this, do that’	*“they might tell him off or they might shout at him…he doesn’t feel safe if people do that”*	*N/A*
Weather	2 (9.5) [1/50]	0 (0)	Grey cloudy heavy day	*“if it’s kind of a grey cloudy heavy day, I think she’s more tense”*	*N/A*
Going out anywhere	3 (14.3) [2/66.7]	1 (11.1)	Being out of the house, not used to being in community, even just leaving the house	*“we want to try and get him out in the community but that does cause him a lot of anxiety”*	*“even just leaving the house for example, all that uncertainty”*
People coming into house to look after her/him	2 (9.5) [0/0]	0 (0)	He wouldn’t accept people coming in, invading his territory	*“if there are new people coming in then that makes her anxious”*	*N/A*
Big people/big men	1 (4.8) [0/0]	0 (0)	Victim of attack	*“he was the victim of a sustained vicious attack so he has got…fear around big men”*	*N/A*
Separation anxiety	2 (9.5) [1/50]	0 (0)	If parents go out of sight when with them, following, clinging	*“going away from me, he feels separation anxiety”*	*N/A*
People talking about individual when they’re present	1 (4.8) [0/0]	0 (0)	Having a conversation with the staff and he’s sat there, talking about the past	*“you’re talking about the past…they are talking around him and he’s listening”*	*N/A*
People suddenly coming into home	1 (4.8) [0/0]	0 (0)	Classing that as his safe place, it’s his home and he’s got somebody from next door coming into it	*“he wouldn’t accept people coming in here… I think its invading his territory”*	*N/A*
Significant life changes	2 (9.5) [0/0]	2 (22.2)	Bereavement, family member/friend moving away	*“she went off to university two years ago so obviously that has had an impact on him as well”*	*“significant loss for some of our youngsters”*
Waiting	2 (9.5) [2/100]	2 (22.2)	He won’t wait for things, wait 3 h before a procedure	*“he finds waiting really hard… if we have to queue or pause in traffic”*	*“he won’t wait for things either”*
Anxious network	0 (0)	2 (22.2)	Parental anxiety, anxiety in the network around an individual	*N/A*	*“increase in anxiety in the network which then might translate to the poor person if they’ve got an anxious network around them”*
Attachment difficulties	0 (0)	3 (33.3)	Didn’t form an attachment with anyone, breakage of primary attachment relationships, individuals going into care, looked after children	*N/A*	*”what we see clinically is the breakage of the primary attachment relationships with people in staff or sometimes parents or carers…that causes increased anxiety”*

#### Clinician Assessment of Anxiety and Challenges of Assessment

To fulfil the second and third aims of the study, further analysis was conducted on clinician interviews to gain understanding of clinicians’ experience of the assessment of anxiety in ID clinical services and the challenges of assessment. IPA was chosen due to its ability to provide an in-depth exploration and sense-making of experiences, in a purposive and relatively homogeneous sample (Smith et al., 2009) (Table 7).

**Table 7:** IPA analysis: final theme table

Themes	Subthemes	Subthemes
Methods of assessment for anxiety	Current anxiety assessment	
	The importance of informant involvement and gathering information across contexts	
Identification of behavioural change	Identification of an individual’s baseline behaviour	
	Importance of knowing an individual well, regular assessment and individualised approach	
Differentiating anxiety from other forms of distress	Behavioural overlap and behavioural divergence	Importance of context and identification of anxiety trigger
	The importance of ruling out other causes of distress	
	Importance of working as part of a multidisciplinary team	
Additional diagnoses	Consideration of diagnoses and impact on presentation	
	Diagnostic overshadowing	

For the IPA analysis, interview coding followed a step-by-step procedure as described by Smith and Osborn (2003). The first transcript was analysed using the following steps: (*i.)* the transcript was read a number of times and initial comments of interest were made; (*ii.)* initial comments were developed into theme titles; (*iii.)* themes were collated and connections were made between themes, enabling more theoretical ordering where the aim was to make sense of theme connections; (*iv.)* as themes and theme connections developed, the transcript was re-visited to ensure that the themes made sense for the words used by the clinician; (*v.)* the themes were then ordered into a table, with the identification of overarching superordinate themes from the transcript. The table from the first transcript was used as a template for subsequent analysis of further transcripts, with careful consideration to ensure new experiences were acknowledged. Once each transcript had undergone this process, a final table of themes was created. To reduce bias and increase validity, a second author (JT) reviewed a subset of the interview transcriptions and theme tables (*n* = 3) to check agreement. Discussion between two authors (GE & JT) led to consensus of the theme tables for the subset of interviews and facilitated the development of the final theme table for all interviews; the process taken was similar to previous IPA studies (Howard et al., 2021; MacMahon et al., 2015).

## Results

### Presentation of anxiety: Content analysis

Across the parent/carer and clinician interviews, the overarching themes of behaviours associated with anxiety and anxiety triggers yielded 22 and 37 codes respectively (Tables 5 and 6). For behaviours associated with or attributed to anxiety, the most frequently reported by parents/carers were increased vocalisation or communication (*n* = 20; 95.2%), behaviours that challenge (*n* = 19; 90.5%) and avoidance/refusal patterns (*n* = 19; 90.5%). Behaviours that challenge included behaviours such as pushing, biting or hitting others (*n* = 13; 61.9%), self-injurious behaviour (e.g. hitting, biting, picking or pinching oneself; *n* = 14; 66.7%) and damage to property (e.g. throwing, kicking or breaking objects; *n* = 10; 47.6%). Behaviours were similar across autistic children and adults; for children, increased vocalisation (*n* = 9, 100%) and behaviours that challenge (*n* = 9, 100%) and for adults, increased vocalisation (*n* = 11, 91.7%) and avoidance (*n* = 11, 91.7%) were most frequently endorsed. Examples of the most frequently reported behaviours with their corresponding code are highlighted in quotes below, with further examples and child vs. adult break-down provided in Table 5. Presentations for each autistic individual are presented in the Supplementary material.

"he might start making loud noises…a groaning" (Mother of 7-year-old male; quote coded as increased vocalisation/communication).

"what he does when he gets anxious, he tries to hit his head, he was kicking out and thumping, when he gets to a certain point, he starts to lash out…throwing chairs all the time" (Mother of 44-year-old male; quote coded as behaviours that challenge).

“not wanting to eat her dinner, or running out.” (Mother of 26-year-old female; quote coded as avoidance/refusal patterns).

For the clinician interviews, the most frequently reported behaviours associated with or attributed to anxiety were behaviours that challenge (*n* = 9; 100%), avoidance or refusal patterns (*n* = 9; 100%) and increased vocalisation or communication (*n* = 8; 88.8%). Behaviours that challenge included behaviours such as pushing, hitting, or pulling hair of others (*n* = 9; 100%), self-injurious behaviour (*n* = 9; 100%) such as hitting or biting oneself and damage to property (*n* = 5; 55.6%) such as hitting or breaking objects.

“lots of anxiety that puts themselves and others at risk, the anxieties they’re presenting cause them to lash out, extreme self-injurious behaviours” (Clinical Nurse Specialist/Lead, C008; quote coded as behaviours that challenge).

“avoidance of doing something they would normally do, a young person trying to refuse to do something, maybe refusing to go to school or refusing to go out” (Clinical Psychologist, C005; quote coded as avoidance/refusal patterns).

“they are increasingly distressed and making increased vocalisations” (Consultant Psychiatrist, C007; quote coded as increased vocalisation/communication).

The most common anxiety triggers reported by parents/carers were unpredictable/uncertain situations and changes to routine (*n* = 16; 76.2%). Young children and animals were reported as triggers of anxiety due to unpredictability. Parents/carers also reported anxiety triggers such as loud noises/particular noises (*n* = 14; 66.7%) and sensory overload (*n* = 14; 66.7%). Most parents/carers (*n* = 14; 66.7%) also reported setting events (defined as broader, background antecedent conditions that are temporally distinct from stimuli that immediately proceed an event; Nosik & Carr, 2015). Examples of setting events were tiredness, hunger and pain/discomfort or physical illness. Triggers for anxiety were explored across autistic children and adults; for children, unmet needs (*n* = 6, 66.7%) and setting events (*n* = 6, 66.7%) were common triggers whilst for adults, unpredictability/uncertainty (*n* = 11, 91.7%) and changes to routine (*n* = 11, 91.7%) were most frequently endorsed. Examples of the most commonly reported triggers with their corresponding code are highlighted in quotes below, with further examples provided in Table 6 with the break-down across children vs. adults.

“it is all to do with things that are unknown, or it can be things that are familiar, but she is just uncertain about it…she doesn’t like change, she likes to have a routine and if something is different then that would upset her” (Mother of 23-year-old female; quote coded as unpredictability/uncertainty and change to routine/expectation).

“if he is hungry, if he is thirsty, if he needs to go to the toilet, if he has maybe constipation and he cannot tell us about it, or the opposite, he has diarrhoea or has some stomach problem” (Father of 11-year-old male; quote coded as setting events).

Clinicians also commonly endorsed anxiety triggers related to changes to routine or expectations (*n* = 9; 100%), followed by unpredictable or uncertain situations/feelings (*n* = 7; 77.8%), setting events (*n* = 7; 77.8%) and specific places, situations, or stimuli (e.g., hospitals, balloons; *n* = 7; 77.8%).

“changes in the everyday routine structures, summer holidays, our referrals do not go up then but crisis calls come through in the summer holidays because I’m guessing the change in routine” (Clinical Psychologist, C005; quote coded as change to routine/expectation).

“Dogs, unpredictability probably, dogs bite. They can’t control a dog; they don’t know what that dog’s going to do” (Community Learning Disability Nurse, C004; quote coded as unpredictability/uncertainty).

### Clinician experience of anxiety assessment: IPA analysis

The IPA analysis conducted on the clinician interviews yielded four themes: methods of assessment for anxiety; identification of behavioural change; differentiating anxiety from other forms of distress; and additional diagnoses (See Table 7 for the final theme table).

#### Theme 1: Methods of assessment for anxiety

This theme describes current assessment methods used by clinicians to assess anxiety. Data within this theme were further categorised into two subthemes: current anxiety assessment and the importance of informant involvement and gathering information/evidence across multiple contexts.

Clinicians described methods to assess anxiety including behavioural records, standardised questionnaires, observations, and Antecedent-Behaviour-Consequence (ABC) charts (record forms used to document events that occur before and after the behaviour of interest is observed; Delgado et al., 2017). However, clinicians noted the overlap between items on measures of other forms of distress such as pain and anxiety questionnaire measures, as highlighted in the quote below.

“what we often find is most of our children score on pain measures… I often wonder whether the overlap is just still too great on these measures… often the child scores highly on everything and then you’re left with that” (Clinical Psychologist; C001).

In keeping with the findings of the content analysis which identified unusual fears, anxiety associated with changes in routine and behaviours that challenge, clinicians also spoke of ‘atypical’ presentations of anxiety. Existing measures may miss these ‘atypical’ presentations, precluding a comprehensive assessment of anxiety.

“We’ve got flying things, pigeons was one… balloons, because they didn’t like the movement of the balloon… didn’t like the texture and the noise and it could pop”. (Community Learning Disability Nurse; C004).

Throughout the interviews, clinicians highlighted the importance of involving informants who know the person well during assessments of anxiety. Such input assisted clinicians in building a picture of how anxiety presents across settings and situations. An informant may be able to identify subtle differences in presentation when an individual is experiencing anxiety versus another form of distress and provide key information about the contexts most likely to induce anxiety related behaviours.

“observations… at home, at school, parent-reporting, school-reporting, reporting from wherever the child is or exists” (Community Learning Disability Nurse; C002).

#### Theme 2: Identification of behavioural change

Clinicians discussed assessment of behaviour change as a key area of importance in the assessment of anxiety. In the clinicians’ opinion, understanding behaviour change is dependent on two factors: being able to identify an individual’s baseline levels of behaviour; and a thorough and individualised approach to assessment leading to in-depth understanding of the individual.

Clinicians stressed the importance of having a working knowledge of an individual’s baseline, including their presentation when they are comfortable, content, and relaxed, to be able to identify changes in behaviour that might indicate distress. This is particularly important as clinicians identified how behavioural markers of distress can overlap with autistic characteristics; for example, repetitive behaviours such as rocking or adherence to routine. Such behaviours may be characteristic of an individual when they are happy and content but may increase in duration or intensity when an individual is feeling anxious.

“What will make you think of anxiety is if there’s been a behavioural change of some sort so that could be anything, it would be thinking about what a baseline of behaviour was for that young person and thinking about clinically what’s changed”. (Clinical Psychologist; C001).

To aid this assessment of behaviour change, clinicians indicated the importance of knowing an individual well, having regular assessments and an individualised approach to assessing anxiety. This subtheme was crucial to helping clinicians identify a change from an individual’s baseline. For example, similar to the importance of informant involvement in the assessment of anxiety, many clinicians commented on individual presentations that became apparent after they got to know an individual, including patterns of behaviour linked to key anxiety triggers. Clinicians commented that it is difficult to assess behaviour when they do not know an individual well; nuances and subtleties of behaviour can be missed. For example, one clinician mentioned that vocalisations can sound the same when an individual is not well-known to them. Several clinicians also highlighted that every individual is different, stressing the importance of an individualised approach to assessing anxiety. Clinicians commented on the importance of spending time with an individual, learning about them, and having regular appointments, including some in the community.

#### Theme 3: Differentiating anxiety from other forms of distress

Theme three includes three subthemes: behavioural overlap and behavioural divergence; the importance of ruling out other causes of distress; and the importance of working as part of a multidisciplinary team.

Speaking from their experience, clinicians reported that behaviours could be present for various reasons and behavioural overlap is common, making it difficult to discern why behaviours may be present. Further complicating this overlap, clinicians described links between factors such as anxiety and pain. For example, clinicians indicated that anxiety can be associated with the experience of physical symptoms (stomach-aches and feeling unwell), and vice versa; whereby pain can be associated with feelings of anxiety.

“it’s really difficult in that sense to determine actually ‘is he anxious?’, ‘is he in pain?’ or is it ‘he’s anxious cause he’s in pain?’ or ‘actually is he not in pain?”. (Community Learning Disability Nurse; C004).

However, most clinicians were able to identify ways that helped them to differentiate the cause of behaviour, allowing behavioural divergence. For example, many mentioned that the presentation of self-injurious behaviour restricted to a particular part of the body (e.g., ear, teeth) was a key indicator of localised pain. Additionally, an acute presentation of a new behaviour, out of the blue and not linked to a known anxiety trigger was also noted as a sign of potential pain. A couple of clinicians explored this in more depth and identified behaviours highlighted in the quote below, with another clinician suggesting that they believed the presentation of aggressive behaviours was less associated with pain and an increase in repetitive behaviour was more likely to be associated with anxiety than pain. Whilst clinicians were able to comment on behaviours that they use to differentiate anxiety from pain, they felt identifying behaviours that differentiate anxiety from low mood or depression was more difficult.

“there’s subtle differences, you see more writhing behaviours in pain as opposed to (anxiety), you might have all the negative vocalisations, being uncooperative, cranky… but you might also see arching of the back, writhing, which for me are quite key related behaviours of pain as opposed to anxiety” (Clinical Psychologist; C001).

Context is also an important factor that clinicians described to help them differentiate anxiety from other forms of distress; this included identifying triggers for anxiety as part of the assessment. Clinicians highlighted how successful identification of an anxiety trigger can aid formulation and inform intervention, whilst the absence of an identified trigger provoked challenges in assessment and subsequent care planning.

“once you have a set of triggers for that person, it would become easily anxious and set off by, I think it would be more easy to pick up on that”. (Clinical Nurse Specialist; C003).

Clinicians consistently stressed the importance of ruling out other causes of distress in individuals who speak few or no words to be more confident that anxiety is the presenting difficulty. For obvious reasons, clinicians particularly highlighted the importance of ruling out pain and physical health conditions during the first phase of assessment. Furthermore, one clinician described drastic, almost immediate changes in behaviour once health-related difficulties were addressed for a particular individual.

“We’d tend to try and rule out pain in the first instance, particularly if the presentation is self-injury… because we know health conditions are more likely in our children and often do get missed… also on ethical grounds… if the child’s in pain, you could do something about it quite quickly”. (Clinical Psychologist; C001).

However, clinicians highlighted that being able to rule out other causes of distress is not always straight-forward and there may be more than one factor contributing to presenting behaviour, which may include the interaction between health difficulties and anxiety.

“I think with a lot of children that we see it (pain) gets dismissed because it’s too difficult to do the investigation on them”. (Consultant Neuropsychiatrist; C006).

To properly assess physical health difficulties and the possibility of pain, clinicians highlighted the importance of working with physical health services/colleagues as part of a multidisciplinary team. There was a sense that this can take substantial time and effort but is essential to ensure the best health and behavioural outcomes for individuals who speak few or no words.

“for us I think that involves a lot of liaising, so say if they were under a paediatrician or school doctor, we’d be liaising with them, just to see what has been done, what could be done”. (Community Learning Disability Nurse; C002).

#### Theme 4: Additional diagnoses

Theme four focuses on the importance of acknowledging additional neurodevelopmental, genetic, or neurological diagnoses and their impact on the identification and assessment of anxiety.

Specifically, clinicians mentioned diagnoses of autism, genetic syndromes, ADHD, and epilepsy. Clinicians stated that identification and diagnosis of conditions, such as autism, can aid understanding of phenotypic factors which inform assessment and formulation. For example, for autistic individuals, clinicians mentioned anxiety triggers such as transitions, routine changes, and sensory processing differences. However, for some rare genetic syndromes (e.g., Fragile X, Klinefelter’s, Prader-Willi, Smith-Magenis and Kleefstra syndromes), a lack of awareness of phenotypic presentations can further complicate mental health assessments.

“the genetic syndrome means they’re more likely to have particular triggers to things and that often will get missed… people still don’t know enough about them or they don’t know to look for specific things”. (Clinical Psychologist; C001).

Many clinicians explored the issue of diagnostic overshadowing; whilst it is important to acknowledge an individual’s comorbid diagnoses and the impact they may have on an individual’s presentation; behaviour must not be assumed to just be part of an existing diagnosis. A thorough exploration of the potential role and impact of anxiety is needed when investigating presenting behaviour.

“…may not explore other things, so I think sometimes it’s tricky because as a default what comes first in line in terms of behaviour, the child’s got learning disability and ASD, could be attributed to that”. (Consultant Psychiatrist; C007).

## Discussion

To our knowledge, the current study is one of the first to focus exclusively on exploring anxiety in autistic individuals with moderate-profound ID who speak few or no words, addressing an identified gap in the literature (Adams & Oliver, 2011; Flynn et al., 2017; Simpson et al., 2020). The study utilised a bottom-up approach, free from the reliance on diagnostic criteria, and collated information from both parents/carers and clinicians, the latter a particular strength as research exploring clinician perspective is scarce (Brookman-Frazee et al., 2012). The clinician interviews provide an overview of the types of methods that can be utilised in clinical practice for assessing anxiety, and the factors that may enhance health and behaviour outcomes. The interviews also gave rise to several considerations that may be important for the development of new assessment measures of anxiety in individuals who speak few or no words (See Table 8 for summary).

**Table 8:** Considerations for new assessment tools

Considerations for the development of new assessment measures	
Explore similar behaviours and triggers to existing measures but develop and validate new measures specifically for individuals who speak few or no words	
Existing measures should be used to pinpoint key behavioural markers of anxiety, otherwise they should be used cautiously	
Spend time getting to know an individual and collate information from several informants across contexts alongside use of questionnaire measures	
Explore the potential role of physical discomfort/pain, form and maintain relationships with colleagues in physical health settings (e.g., MDT formulation)^a^ to achieve this	
Incorporate the assessment of baseline presentation to identify behavioural change and help tackle diagnostic overshadowing	
Consider comorbid diagnoses and the impact they may have on an individual’s presentation	

^a^Multidisciplinary team

The study used a two-stage approach to quantify behaviours that may be associated with anxiety and clinician experiences of anxiety assessment. Parents/carers and clinicians attributed similar behaviours to anxiety, including behaviours that challenge and increased vocalisation. This is consistent with studies using questionnaire and behavioural observation measures that indicate autistic individuals with ID and those with language difficulties may show these behaviours when anxious (Bitsika et al., 2016; Moskowitz et al., 2013). Avoidance/refusal behaviours were frequently endorsed by parents/carers and clinicians, a finding consistent with the typically developing anxiety literature (Dymond & Roche, 2009; Hofmann & Hay, 2018).

Parents/carers and clinicians reported similar triggers for anxiety, such as unpredictable situations/feelings and changes to routine. These findings concur with existing research identifying ‘atypical’ anxiety in autistic individuals without ID or mild ID, who speak in sentences (Bearss et al., 2016; den Houting et al., 2018; Kerns et al., 2016; Vasa et al., 2016). Existing research highlights associations between anxiety and intolerance of uncertainty in autistic individuals, which is consistent with unpredictable situations/feelings in the current study (Boulter et al., 2014; Jenkinson et al., 2020).

Four key themes emerged from the interviews with clinicians about their experiences of assessing anxiety; (1) methods of assessment for anxiety, (2) identification of behavioural change, (3) differentiating anxiety from other forms of distress and (4) additional diagnoses. First, clinicians commented on the limitations of existing methods of assessment. This mirrors limitations of existing tools reported in previous research: reliance on traditional diagnostic criteria and limited consideration of the overlap of anxiety with other forms of distress such as physical discomfort or pain (Flynn et al., 2017; South et al., 2017; Tarver et al., 2020a). As autistic individuals and individuals with ID are at increased risk of physical health difficulties and experiencing discomfort/pain, clinicians perceiving this limitation in existing measures is particularly concerning (Doody et al., 2017; Whitney & Shapiro, 2019). As also indicated in previous research, clinicians in the current study highlighted the importance of informant involvement from someone who knows the individual well and collating information from different sources across contexts when assessing anxiety (Spain et al., 2017; Vasa et al., 2016; White et al., 2009). Linking to this is the importance of multi-method assessment of anxiety which may include use of psychophysiological measures, due to the identified limitations of other forms of assessment. There is evidence of reduced heart rate and cortisol responsiveness relating to anxiety in autistic individuals (Hollocks et al., 2014). Furthermore, Ferguson et al. (2019) has demonstrated the feasibility of psychophysiological methods, whereby increases in electrodermal activity preceded behaviours that challenge in autistic individuals with ID. Further research into the acceptability and feasibility of such measures in this population is crucial to complement other methods of anxiety assessment (Ferguson et al., 2019; Hollocks et al., 2014).

Second, clinicians commented on the importance of identifying an individual’s baseline behaviour to help pinpoint behavioural change that may be associated with anxiety, a finding consistent with previous research (Ozsivadjian et al., 2012; Tarver et al., 2020a). Identifying an individual’s baseline behaviour may help to discern which behaviours are ‘typical’ for that individual, helping to disentangle autism characteristics from anxiety (Kerns & Kendall, 2012; van Steensel & Heeman, 2017; Vasa et al., 2016, 2018). Developing and validating assessments that address the overlap between autism characteristics and anxiety is a top priority to improve anxiety assessment in individuals who speak few or no words (Vasa et al., 2018).

In the third theme, clinicians explored behavioural overlap between anxiety and other forms of distress, such as pain. Investigating and ruling out other potential causes of distress, as part of a multidisciplinary team, was one way that clinicians aim to identify anxiety; an approach which is consistent with National Institute of Health and Care Excellence (NICE, 2013) guidelines. Furthermore, clinicians discussed the importance of context for identifying anxiety triggers; once triggers were identified, clinicians felt able to support individuals through to discharge from a service.

Finally, within theme four, clinicians emphasised the importance of considering additional diagnoses such as genetic syndromes, ADHD, and epilepsy, due to evidence of high rates of comorbidity with autism and ID (Karam et al., 2015; Neece et al., 2011; Robertson et al., 2015; Tonnsen et al., 2016). Gordon-Lipkin et al. (2018) found autistic individuals with ADHD are at greater risk of experiencing anxiety than autistic individuals without a diagnosis, therefore ADHD may be implicated in the development and presentation of anxiety. Furthermore, genetic syndromes are associated with specific presentations of anxiety. For example, in Williams Syndrome, phobias related to auditory stimuli are commonly reported and have been associated with hyperacusis, and the deletion of GTF2I is associated with low rates of social anxiety (Dykens, 2003; Klein et al., 1990; Procyshyn et al., 2017; Royston et al., 2017). Clinicians should explore how phenotypic characteristics may interact with and have an impact on the presentation of anxiety (Waite et al., 2014). Also, within the final theme, the ongoing challenge of diagnostic overshadowing was discussed, as evidenced in previous research (Kerns et al., 2015; Mason & Scior, 2004). Anxiety is distinguishable from other diagnoses and therefore assessment measures need to pinpoint the role and impact of anxiety on an individual, to allow targeted intervention to improve quality of life (Kerns et al., 2016; Renno & Wood, 2013).

More widely, the interviews provide a helpful summary to facilitate further learning for people who are new to the field of ID. The behaviour presentations obtained from parent/carer and clinician responses provide a starting point for identifying key patterns in behaviour when anxiety is considered a difficulty. These presentations could be disseminated through clinical training programmes to facilitate discussion around the potential for overlap and confusion with signs of other difficulties (e.g., pain), as highlighted in this paper.

It is important to note the limitations of this study. Firstly, whilst the clinician sample size was appropriate for the in-depth IPA analysis, the study explored views of clinicians in one geographic location. The current assessment methods and identified challenges of assessment may not be representative of clinicians working in other services. Therefore, while these findings may facilitate discussion and generate considerations for assessment design, service delivery or follow-up studies, it is not suggested that our findings should be implemented within clinical practice without reflection and further assessment, and substantial changes to practice based on these findings alone would be premature. Second, while the behaviours described may be useful for generating items for new anxiety measures, the behaviours are only based on parents/clinicians’ attributions about anxiety. Further research will be needed to validate items included in new assessment measures to ensure these items are capturing anxiety. Thirdly, within qualitative research it is recommended that researchers obtain respondent validation (obtaining feedback from participants to ensure the analysis, interpretation and conclusions drawn align with their views; Busetto et al., 2020). This was beyond the scope of the current study as included participants were asked to provide feedback on the developed measure, currently undergoing validation. The decision not to obtain respondent validation could be considered a limitation of the study, but was justified to avoid overburdening research participants. Finally, future research could adopt an approach whereby interviews are completed with parents/carers and clinicians about the same autistic individual, to allow triangulation of data which may help to discern whether an individual’s presentation is due to anxiety or another cause of behaviour.

### Implications

The current study has key implications for researchers and clinicians. For researchers, when developing new assessment tools, teams may wish to take note of the considerations and challenges discussed by clinicians to ensure that assessment tools meet the needs of those likely to use them, and the populations they are designed to assess. Involving individuals, parents/carers and clinicians in the development of such assessment tools is crucial to achieve this. For example, the current study highlighted change from baseline as a key factor to identify behavioural change, existing measures such as the ASC-ASD and the GAS-ID do not appear to explicitly assess this (Bearss et al., 2016; Mindham & Espie, 2003; Rodgers et al., 2016). We would recommend that further iterations of existing measures or in the development of new measures for autistic individuals with ID that strategies are implemented to assess change from baseline.

Furthermore, the current study highlighted that similar behaviours and triggers associated with anxiety are identified across autistic children and adults. There was indication that unmet needs were more commonly endorsed for autistic children, this may be due to adults developing communication strategies over time to effectively communicate their needs. Setting events were endorsed at the same rate (66.7%) for both children and adults. Due to the qualitative nature of the study, it was beyond the scope to explore age differences statistically, therefore future research should explore the presentation of anxiety across age groups as well as gender (Adams et al., 2019). It may be that different behaviours and triggers are relevant to different groups, with the possibility of gender differences and/or age-related changes in presentation which will inform early identification and intervention. This would also have important implications for parents/carers responses to the person they care for’s anxiety as this may vary by age and/or gender.

For clinicians, especially those working within mental health and behaviours that challenge pathways, working as part of a multi-disciplinary team is crucial due to behavioural overlap described in the current study. This collaborative, holistic approach should be adopted to rule out other possible causes of distress such as pain. The study also promotes reflection on the current methods of assessment of anxiety and associated challenges in individuals who speak few or no words. In-depth exploration of clinician experience is crucial to ensure that assessment tools are developed addressing the challenges faced within clinical practice. It may be that sharing clinician experience encourages conversation, new approaches to assessment and the formation of strategies to overcome highlighted challenges.

The study highlights the potential vulnerability of autistic individuals with ID who speak few or no words, where identification and assessment of anxiety is not straight-forward. Behaviours that challenge are observed in autistic individuals when appearing anxious, however it may not be considered that anxiety is a potential underlying factor. This has implications for professionals and sectors who individuals with ID may come into contact with (e.g. hospitality, law-enforcement, medical professionals). It is important that professionals have knowledge, ideally training, on how to best support autistic individuals who may be experiencing distress, including identification of a trusted adult who considers the individual’s best interests.

In summary, the current study identified key behavioural markers associated with anxiety, triggers of anxiety and reflections on current anxiety assessment; all of which may be useful to inform the development of new clinical assessment tools for anxiety in individuals who speak few or no words. The feedback from clinicians across multiple themes within the IPA analysis highlighted the need for time and in-depth knowledge of clients to ensure the best health and behaviour outcomes for autistic individuals with ID. Improving the identification and assessment of anxiety will enable clinicians to provide targeted support early, improving quality of life for individuals and their families.

## Supplementary Information

Below is the link to the electronic supplementary material.

Supplementary file1 (PDF 114 kb)
