# Supplementary material for: Utilising Interview Methodology to Inform the Development of New Clinical Assessment Tools for Anxiety in Autistic Individuals Who Speak Few or no Words
Source: J Autism Dev Disord. 2022 Mar 18;53(6):2328–48. doi: 10.1007/s10803-022-05509-y (PMC10229722; doi:10.1007/s10803-022-05509-y)
Supplement: Supplementary file 1 — Supplementary file1 (PDF 114 kb) [file 10803_2022_5509_MOESM1_ESM.pdf]

Supplementary material: Journal of Autism and Developmental Disorders

Utilising interview methodology to inform the development of new clinical assessment tools for anxiety in autistic individuals who speak few or no words

Georgina Edwards*[1], Joanne Tarver[1], Lauren Shelley[1], Megan Bird[1], Jessica Hughes[1], Hayley Crawford[2] & Jane Waite[1]

[1]Department of Psychology, School of Life and Health Sciences, Aston University, UK.

[2]Mental Health and Wellbeing Unit, Warwick Medical School, University of Warwick, UK.

*Correspondence to Georgina Edwards, Department of Psychology, School of Life and Health Sciences, Aston University, B4 7ET, UK. Email: edwardg4@aston.ac.uk

Table 1. Anxiety profiles of participants based on interview schedule

	Participants					
	**1** (19-year-old male)	**2** (26-year-old female)	**3** (23-year-old female)	**4** (11-year-old male)	**5** (26-year-old male)	**6** (44-year-old male)
Setting events		Poor sleep, illness e.g. constipation		Illness e.g. constipation		Illness e.g. operation and constipation
Triggers	Routine changes, new situations, SP of thresholds	Sensory overload	SI, everyday situations, routine changes, new situations, sensory overload,	High demands, sensory overload	SI, new situations, sensory overload, SP of loud noises, dogs, children, big men (violent attack)	Routine changes, sensory overload
Primary responses	Tense muscles, restless, clinging to parents	Not relaxed, agitated, wants control of situations, change in her mood	Rubs arms, hands start to shake/tremble	Crying, covering ears with hands, pushing parents away when comforting, biting between thumb and wrist, easily upset	Vocalisation (rumble), fingers held in front of face, eyes glaze over, walk differently, drop chin to chest, frowns	Body rocking, moving about, waving arms, switches off, throwing self back, body posture changes
Secondary responses	Flee, increased vocalisation, change in appetite, defiance, pacing, tense muscles, clinging to parents	SIB, aggression, repetitive behaviour and speech, tense face, recurrent urge to go to the bathroom, change in appetite, decreased activity, throws objects, tone change, hypervigilant	Tremble, SIB, increased vocalisation, decreased activity, tense muscles, lies down and rubs face on floor/with hands	SIB, aggression, crying, repetitive behaviour, grimacing, increased vocalisation, decreased activity, self-soothing behaviour e.g. swings, tense muscles, irritability, echolalia	SIB, crying, flee, frowns, increased vocalisation, pacing, muscles tense, cover face with hands	Tremble, SIB, aggression, repetitive behaviour, twitching, gets obsessional, decreased or increased vocalisation, sweat, eats less, throws objects, makes self-sick, urge to go to the bathroom, muscles tense

SIB: self-injurious behaviour, SI: social interactions, SP: specific phobia

	Participants					
	**7** (18-year-old male)	**8** (7-year-old male)	**9** (21-year-old male)	**10** (19-year-old male)	**11** (19-year-old male)	**12** (11-year-old male)
Setting events				Illness e.g. gut related problems		
Triggers	Routine changes, new situations, sensory overload, AS, SP of small spaces	SI, routine changes, new stuations, sensory overload, SP of other children	SI, routine changes, sensory overload, SP of young children, unpredictable things	Routine changes, new situations, sensory overload, AS, SP of dogs and children	Everyday events, routine changes, worry about future events, SP of dogs	High demands, everyday events, routine changes, new situations, AS, SP of dogs
Primary responses	Biting self, vocalisations, increase in body movement	Repeated words, looks upset and emotional, body tensing up		Makes a 'shhh' noise, changes in breathing		Repetitive asking, becomes tense, can be really quiet
Secondary responses	SIB, aggression, repetitive behaviour, flee, tense face, increased vocalisation, defiance, pacing, throws objects, hands in ears	Tremble, flee, increased vocalisation, pacing, becomes tense, undresses and tries to get into bath, change in appetite	Changes in breathing, repetitive behaviour, frowning, increased vocalisation, jumpy, decreased activity, pacing, seeks physical comfort	Changes in breathing, repetitive behaviour, less responsive, becomes expressionless, increased vocalisation, nausea, change in appetite, pacing	Palpitations, flee, repetitive speech, sweat, decreased activity, self-soothing, proximity seeking to mum, hopping, refusing to walk	Blushing, becomes expressionless, palpitations, SIB, aggression, crying, repetitive behaviour, flee, throws objects, nausea, changes in appetite, urge to go to bathroom

AS: aversive setting (school, beach, shops), SP: specific phobia, SI: social interactions, SIB: self-injurious behaviour

Participants

	**13** (52-year-old male)	**14** (7-year-old male)	**15** (24-year-old male)	**16** (18-year-old female)	**17** (14-year-old male)	**18** (12-year-old male)
Setting events		Poor sleep		Lack of sleep, constipation and hormonal changes e.g. menstrual cycle		
Triggers	High demands, routine changes, sensory overload, AS, SP of dogs	High demands, everyday events, separation, routine changes, sensory overload, AS, new situations	Everyday events, routine changes, sensory overload, SP of tomato sauce	High demands, separation/being alone, routine changes, sensory overload	New situations, sensory overload, SP of dogs	SI, AS
Primary responses	Say people's names, different names for how anxious they are feeling	Well-up, says certain phrases	Repetitive speech, "no, no, no"	Quiet, withdrawn, angry sounds, bites into scarf, freezing, "responses aren't normal"	Makes sounds, tunefully sings songs, sounds anxious, high pitched, repetitive and louder	Fleeing, looking at parents
Secondary responses	Changes in breathing, repetitive behaviour and speech, opens and closes jaw, increased vocalisation, pacing, throws objects, proximity seeking	Tremble, palpitations, SIB, aggression, crying, repetitive behaviour, flee, looks anxious, increased vocalisation, defiance, throws objects, cling to mum	Blushing, aggression, flee, hands over ears, increased vocalisation, pacing, muscles tense, tone change, faster movements	Changes in breathing, SIB, aggression, crying, tense face, increased vocalising e.g. angry sounds, repetitive speech, defiance, bites things, pulls hair	Changes in breathing, SIB, repetitive behaviour, looks anxious, repetitive speech, jumping on the spot, tone change, reassurance seeking to get needs met	Aggressive towards others, flee, increased vocalisation

AS: aversive setting (dentist, hospital), SP: specific phobia, SIB: self-injurious behaviour

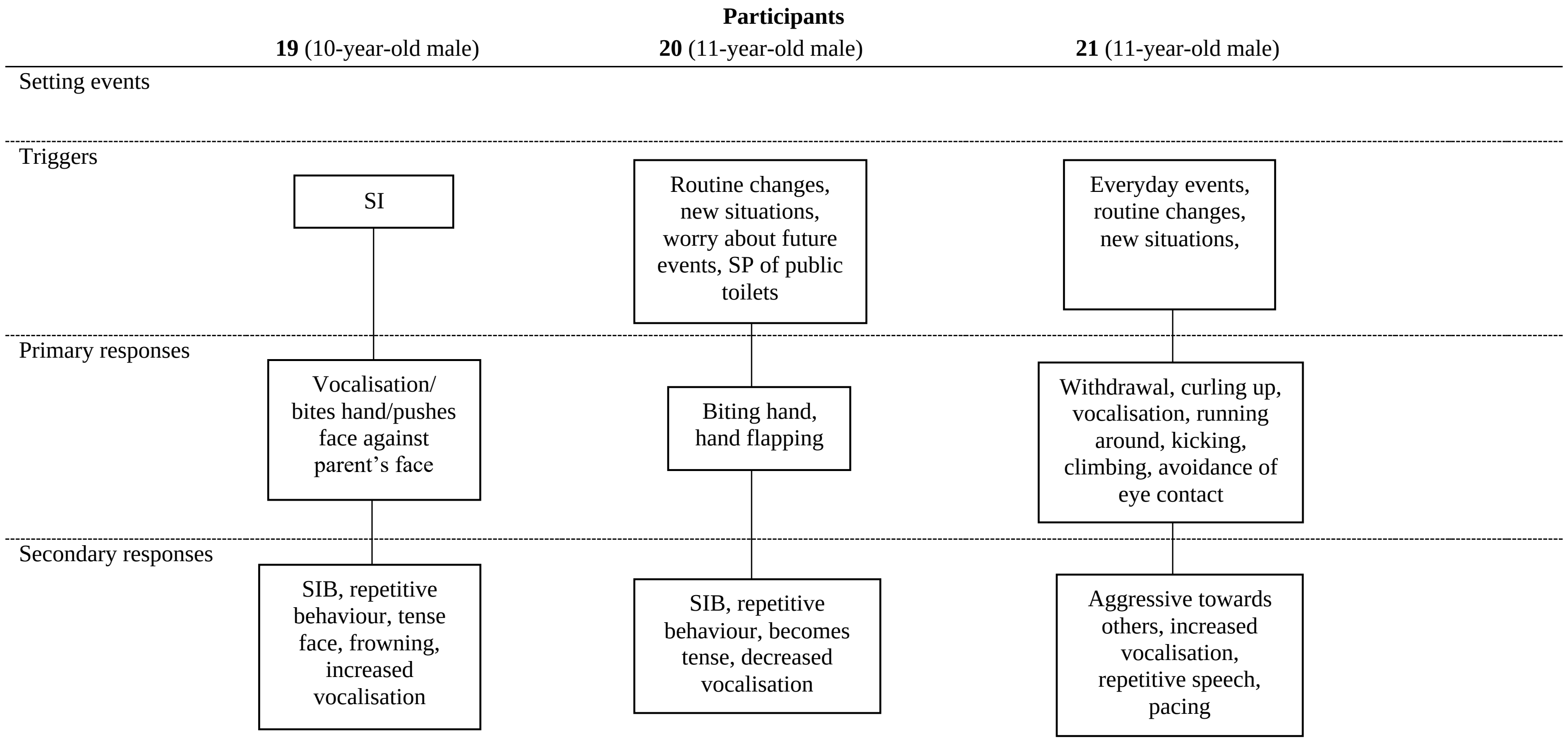

SI: social interactions, SIB: self-injurious behaviour, SP: specific phobia
